# Assessment of Video Quality and Reliability of YouTube Videos Regarding Meniscus Tear Rehabilitation

**DOI:** 10.7759/cureus.36299

**Published:** 2023-03-17

**Authors:** Varag Abed, Matthew Ray, Joseph Smathers, Austin V Stone

**Affiliations:** 1 Orthopaedic Surgery and Sports Medicine, University of Kentucky, Lexington, USA

**Keywords:** online, youtube, meniscus, rehab, physical therapy rehabilitation, meniscus tear

## Abstract

Introduction: YouTube is an open-source platform where creators can record and upload videos for others to see. As the popularity of YouTube increases, it is being increasingly used for healthcare-related information. However, with the relative ease of uploading videos, the content quality of individual videos is not regulated. This study aimed to assess and analyze the content quality of YouTube videos concerning meniscus tear rehabilitation. We hypothesized that most videos would be of low quality.

Methods: The keywords “meniscus tear treatment,” “meniscus tear recovery,” “meniscus tear physical therapy,” and “meniscus tear rehabilitation” were used for searching videos on YouTube. A total of 50 videos was analyzed in this study that related to meniscal rehabilitation, grouped into one of four categories: non-physician professionals (physical therapists and chiropractors) (n=28), physicians (with or without academic affiliation) (n=5), non-academic healthcare-related websites (n=10), and non-professional individuals (n=7). Videos were then assessed by two independent authors using three scoring systems, which included the Global Quality Scale (GQS), modified DISCERN, and Journal of the American Medical Association (JAMA) scores. The number of likes, comments, video length, and views were collected for each video. Kruskal-Wallis tests were used to compare quality scores and video analytics.

Results: The overall median GQS, modified DISCERN, and JAMA scores were 3 (interquartile range (IQR): 2-3), 2 (IQR: 2-2), and 2 (IQR: 2-2), respectively. Sorted by GQS scores, 20 videos were of low quality (40%), 21 were intermediate (42%), and nine were of high quality (18%). Non-physician professionals produced 56% (28 of 50) of the videos assessed, with physical therapists constituting 86% (24 of 28) of this category. The median duration of each video was 6:54 (IQR: 3:59-10:50) minutes, views was 42,262 (IQR: 12,373-306,491), and likes was 877 (IQR: 239-4,850). Kruskal-Wallis testing showed a significant difference between JAMA scores, likes, and video length duration between video categories (p<0.028).

Conclusion: The overall median reliability of YouTube videos on meniscus tear rehabilitation measured by JAMA and modified DISCERN scores was low. The overall median video quality, as assessed by GQS scores, was intermediate. Video quality was highly variable, with fewer than 20% meeting high-quality standards. As a result, patients are often viewing lower quality videos when researching their condition online.

## Introduction

Meniscus tears are a common condition seen by orthopedic surgeons, with an incidence of 61 per 100,000 people in the United States [1]. There has been an increase in the incidence of meniscal injuries, possibly due to increased sport participation and ease of imaging [2]. An estimated 49% of outpatient orthopedic patients utilize the internet to search for their condition before seeing a physician, while 42% search for their condition on the internet after their appointment [3]. With the high prevalence of meniscus tears and the increased accessibility to the internet, patients may be turning to the internet for information about their injuries.

One of the most popular video-sharing platforms on the internet is YouTube [4]. YouTube is an open-source platform where creators can record and upload videos for others to see. As the popularity of YouTube increases, it is being increasingly used for healthcare-related information [5]. However, with the relative ease of uploading videos, the content quality of individual videos is not regulated. As a result, YouTube cannot screen and guarantee the accuracy of each video, potentially exposing patients to lower quality information. The quality of the information provided on YouTube for the rehabilitation of common orthopedic conditions has been previously examined for anterior cruciate ligament reconstruction (ACLR) and medial patellofemoral ligament reconstruction (MPFLR), but meniscus tear rehabilitation remains unexamined [5-7].

The purpose of this study was to assess and analyze the content quality of YouTube videos concerning meniscus tear rehabilitation. We hypothesized that most videos would be of low quality.

## Materials and methods

The keywords “meniscus tear treatment,” “meniscus tear recovery,” “meniscus tear physical therapy,” and “meniscus tear rehabilitation” were used for searching videos on YouTube on August 29, 2022 [4]. YouTube utilizes the users' age, gender, geographic location, and watch history to personalize search results [5]. Therefore, to account for these confounders, we conducted our search using an incognito mode Google Chrome web browser to anonymize our results. We also closed the web browser and cleared the stored cache between each search. By using incognito mode, YouTube was prevented from using personalized search history and variables to influence search results [5]. The first 60 videos from each search term were assessed by two experienced authors in meniscus tear rehabilitation. If there were unresolved disagreements between the two authors, a third (senior) author was consulted. Exclusion criteria included any videos pertaining to surgical intervention, general meniscus tear information without rehabilitation considerations, or non-rehabilitation care, such as stem cell therapy. Duplicate videos, non-English videos, and non-meniscal rehabilitation videos were excluded as well. Following exclusion criteria, 50 videos were analyzed in this study.

Previous studies have stated that a large percentage of YouTube users watch videos from the first three pages of their search results, and as a result, our search should cover most of what YouTube users would see [8]. The remaining videos were then grouped into one of four categories: non-physician professionals (physical therapists and chiropractors), physicians (with or without academic affiliation), non-academic healthcare-related websites, and non-professional individuals.

Quality assessment

Videos were assessed by the Global Quality Scale (GQS) (Table 1), modified DISCERN (Table 2), and Journal of the American Medical Association (JAMA) scores (Table 3). These scoring systems have been used in prior YouTube studies [5, 8-12]. GQS measures video quality content on a 5-point scale as it relates to quality, flow, topics covered, and usefulness [5]. Scores 1-2 indicate low quality, 3 indicate intermediate, and 4-5 indicate high quality [8]. The modified DISCERN score assesses the reliability and accuracy of a video on a 5-point scale. A point is added if a video is concise, reliable, balanced, references sources, and addresses uncertainty. Higher scores indicate greater reliability [13]. JAMA has a maximum score of 4, where a point is added if a video addresses authorship, attribution, disclosure, and currency (dates when content was posted and updated). Higher scores indicate greater reliability [13].

**Table 1:** GQS Description Table adapted from Guneri et al. [14]. One score is picked. GQS, Global Quality Scale

Score	GQS Description
1	Poor quality, poor flow, most information missing, not useful for patients.
2	Generally poor quality and flow, some information given, but of limited use to patients.
3	Moderate quality, suboptimal flow, some information is adequately discussed, somewhat useful for patients.
4	Good quality and flow, most relevant information is discussed, useful for patients.
5	Excellent quality and flow, very useful for patients.

**Table 2:** Modified DISCERN Description Table adapted from Guneri et al. [14]. Each “yes” to a question adds 1 point to the final score.

Score	Modified DISCERN Description
1	Are the video’s aims clear, concise, and achieved?
2	Are valid and reliable sources cited?
3	Is the information discussed balanced and unbiased?
4	Are additional sources of information listed for patient reference?
5	Does the video address areas of controversy and uncertainty?

**Table 3:** JAMA Description Table adapted from McMahon et al. [15]. Each “yes” to a question adds 1 point to the final score. JAMA, Journal of the American Medical Association

Score	JAMA Description
1	Authorship: are author/contributor credentials and their affiliations provided?
2	Attribution: is copyright information listed, and references/sources for content provided?
3	Currency: is the initial date of posted content and dates of subsequent updates to content provided?
4	Disclosure: are conflicts of interest, funding, sponsorship, advertising, support, and video ownership fully disclosed?

Video parameters

The number of likes, comments, video length, and views were collected for each video.

Statistical analysis

The collected data were tabulated and analyzed by Microsoft Excel software (Microsoft Corporation, Redmond, WA). Two reviewers needed to be in agreement for each scoring system prior to analysis. All continuous outcome variables were analyzed for normality using the Shapiro-Wilk test. Since most of these outcomes violated the assumption of normality (along with the desired retainment of outliers), continuous variables were reported using medians and interquartile ranges (IQR). Kruskal-Wallis tests were used to compare quality scores and video analytics. Across all analyses, a p-value of less than 0.05 was considered significant.

## Results

Of the initial 240 videos, 149 met one or more exclusion criteria, resulting in 98 videos that met the inclusion criteria. A random sample of 50 was extracted to be assessed and scored. Overall video statistics are shown in Table 4.

 

**Table 4:** Overall Video Statistics Values are reported as median (interquartile range).

Video Statistic	Value
Duration in Minutes	6:54 (3:59-10:50)
Views	42,262 (12,373-306,491)
Likes	877 (239-4,850)
Comments	63 (11-305)

**Figure 1:**
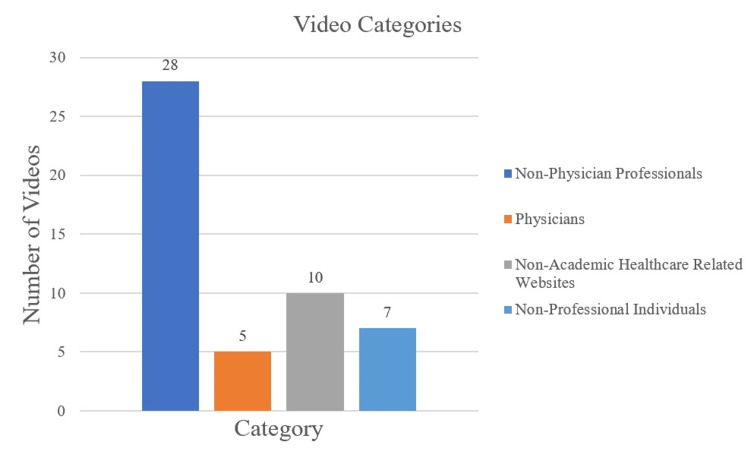
Number of Videos in Each Category

**Figure 2:**
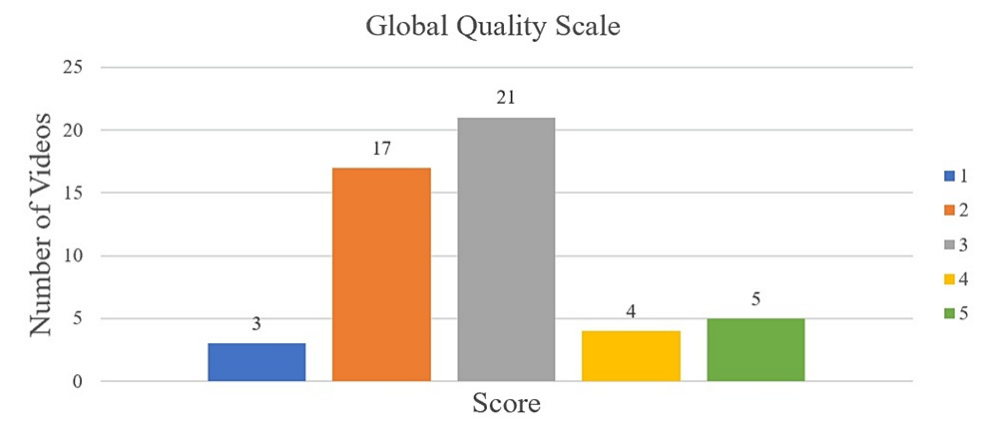
GQS Score Distribution Scores of 1-2 indicate low quality, 3 indicate intermediate, and 4-5 indicate high quality. GQS, Global Quality Scale

**Figure 3:**
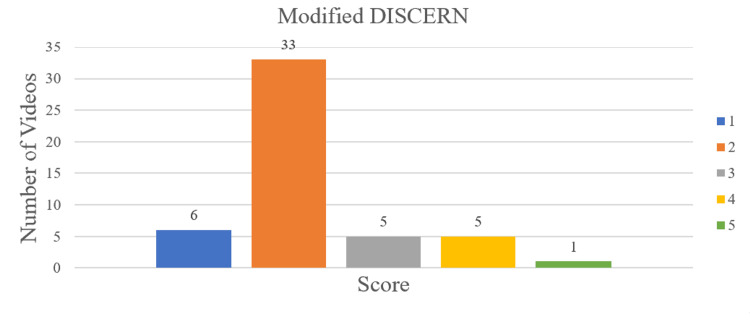
Modified DISCERN Score Distribution Higher scores indicate greater video reliability.

**Figure 4:**
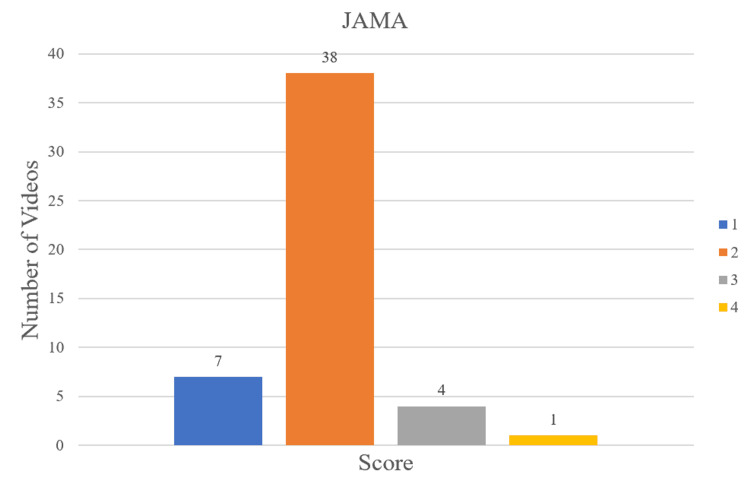
JAMA Score Distribution Higher scores indicate greater video reliability. JAMA, Journal of the American Medical Association

The number of videos in each category is showcased in Figure 1. The distribution of GQS, modified DISCERN, and JAMA scores is displayed in Figures 2, 3, 4, respectively. Median scores for each scale were 3 (IQR: 2-3), 2 (IQR: 2-2), and 2 (IQR: 2-2), respectively. The summary of all statistics broken down by category is shown in Table 5.

According to GQS scores, the median overall quality of videos was intermediate (median=3). A total of 20 videos were of low quality (40%), 21 were intermediate (42%), and nine were of high quality (18%). The median number of views, likes, comments, and duration are shown in Table 5.

**Table 5:** Summary of Video Statistics by Category Values are reported as median (interquartile range). GQS, Global Quality Scale; JAMA, Journal of the American Medical Association

Variable	Overall (n=50)	Non-Physician Professionals (n=28)	Physicians (n=5)	Non-Academic Healthcare-Related Websites (n=10)	Non-Professional Individuals (n=7)
GQS	3 (2-3)	3 (2-3)	3 (3-3)	2.5 (2-3.75)	2 (2-3)
Modified DISCERN	2 (2-2)	2 (2-2)	2 (2-4)	2.5 (2-3.75)	2 (2-2)
JAMA	2 (2-2)	2 (2-2)	2 (2-2)	2 (2-2)	1 (1-1.5)
Views	42,262 (12,373-306,491)	83,366 (16,589-335,506)	58,104 (47,532-74,785)	29,244 (10,767-448,240)	25,509 (10,295-51,024)
Likes	877 (239-4,850)	2,000 (374-6,225)	530 (499-706)	586 (145-1,525)	796 (195-1,400)
Comments	63 (11-305)	107 (14-368)	22 (18-50)	20 (6-115)	102 (35-195)
Duration (Minutes)	6:54 (3:59-10:50)	7:32 (4:58-11:30)	4:19 (3:37-4:51)	3:32 (2:54-4:49)	10:14 (7:32-20:24)

Kruskal-Wallis tests comparing GQS scores, modified DISCERN scores, views, or comments separated by the four video categories showed no significant differences. However, there was a statistically significant difference in JAMA scores (p=0.028), number of likes (p=0.019), and video length duration (p=0.015) between the four video categories.

## Discussion

Our study found that the overall median reliability of YouTube videos on meniscus tear rehabilitation measured by JAMA and modified DISCERN scores was low. The overall median video quality, as assessed by GQS scores, was intermediate. However, far more videos were of low quality (40%) than high quality (18%), potentially exposing patients to incomplete or lower quality information. Non-physician professionals produced 56% (28 of 50) of the videos assessed, with physical therapists constituting 86% (24 of 28) of this category. Chiropractors constituted the remaining 14% (4 of 28). Physician representation was minimally present, as only 10% of videos were produced by them. There was a significant difference between JAMA scores, likes, and video length duration between the four video categories.

There have been several YouTube studies assessing the quality of various medical content, with most showing that the quality is generally poor [6, 16-21]. YouTube does not regulate the content its creators provide and provides search results based on a user’s personalized search history, age, gender, and geolocation rather than the highest quality videos [5]. Assessing video length is an important aspect, as it has been shown that there is a significant drop in engagement if a video is longer than 2 minutes and another if it is longer than 12 minutes [5]. Our overall median video length was found to be 6:54 minutes, which is long enough to provide information but short enough to retain user engagement.

With the nature of searching for rehabilitation videos, it is expected that physical therapists have the greatest number of videos posted. Almost half of the videos (24 of the 50) were made by physical therapists. Physicians, contrarily, produced only 10% of videos. The non-physician professional and physician categories had higher median JAMA scores than non-professional individuals. An explanation for this finding can be that physicians and physical therapists are more likely uploading evidence-based information, while non-professional individuals are more likely to upload anecdotal information based on their experience.

Langford and Loeb assessed the association between perceived patient-provider communication quality and sociodemographic factors by watching YouTube health-related videos. It was found that patients who had a higher perceived quality of patient-provider communication had lower odds of watching health-related videos on YouTube [18]. As a result, if patients do not feel that their providers spend adequate time explaining health-related information to them or allow them to ask questions, they are more likely to turn to online sources. Since YouTube has shown to be a poor source for general meniscal information, and our study showcases it as a poor source for meniscus tear rehabilitation, it is an unreliable source for medical information on meniscus injuries and management [22]. Not only is YouTube a poor source for meniscal information, but it has also been shown to be a poor source for hip arthroscopy, ACLR, or total hip replacement information [6, 16, 23].

Kunze et al. evaluated the reliability and educational content of YouTube videos regarding menisci; however, they did not specifically look into rehabilitation videos like our study. They found that the mean video duration was 9:11 minutes and had 288,598 views. Mean JAMA and GQS scores were 1.55 and 2.12, respectively. Non-physicians were the most common content creators (24%), and videos mainly concerned disease information (38%). JAMA scores were significantly higher in videos uploaded by physicians compared to others [22]. Our study builds upon theirs, as we specifically focused on rehabilitation videos. Overall, JAMA and GQS scores were similar between our studies.

As the use of the internet grows, patients are more and more likely to search for their conditions online. It may be beneficial for orthopedic providers and physical therapists to create their own video-sharing platform or YouTube channels with a target audience aimed toward their patients. In our search, physical therapists produced the greatest number of videos (48%, 24 of 50) due to the nature of searching for rehabilitation videos. They can produce high-quality videos on the platform to later be shared with patients. In the event that patients would like to gain more information on meniscus rehabilitation outside of the limited time they have with their orthopedic provider or physical therapist, they can watch the online videos at their leisure.

This study was not without limitations. Since this was a cross-sectional online study, more videos were likely added related to meniscus tear rehabilitation since our search. Also, there could be a variation in how YouTube recommends videos to its users. While the scoring systems used have definitive requirements for scores, individual video scoring is intrinsically subjective. We attempted to mitigate this by having two authors be in agreement for each score. Finally, only English language videos were assessed.

## Conclusions

The overall median reliability of YouTube videos on meniscus tear rehabilitation measured by JAMA and modified DISCERN scores was low. The overall median video quality, as assessed by GQS scores, was intermediate. Video quality was highly variable, with fewer than 20% meeting high-quality standards. As a result, patients are often viewing lower quality videos when researching their condition online.
